# 

**Published:** 1884-12

**Authors:** 


					﻿DECEMBER, 1884.
b^Ql.O SERIE
, v'li W.
X^L/S^
<? 1855 y>
NEW SERIE?]
,Vol. I - No.»10 7 f
Mm
SWW*	AZa V^XSC
• jDURNA^S.
rVpW by* W.F.WESTMORECAND.M.IJ.? z
AkA H.V.M.MILLER,M.D.LL.D.^Lf .
ML 7%-^	JAMES A.GRAY, M.D."W
W 6---------J
v/uBLISHED By JAMES P.HARRISON&C®S|
_______________Atlanta ,ga. ^gB
SUBSCRIPTION: 2.50 A YEAR.
				

